# Uso de herramientas predictivas en el manejo de pacientes COVID-19: el papel fundamental de los laboratorios clínicos

**DOI:** 10.1515/almed-2021-0019

**Published:** 2021-03-19

**Authors:** Carla Martín Grau, Clara Benavent Bofill, Ester Picó-Plana, Gemma Recio Comí, Margarida Terrón-Puig, Natalia Bastón Paz, MaTeresa Sans Mateu, Cristina Gutiérrez Fornés

**Affiliations:** Laboratorio de Análisis Clínicos, Instituto Catalán de Salud (ICS)- Camp de Tarragona-Terres de l’Ebre, Hospital Universitario Joan XXIII, Tarragona, España; Laboratorio de Análisis Clínicos, Instituto Catalán de Salud (ICS)- Camp de Tarragona-Terres de l’Ebre, Hospital Verge de la Cinta Hospital de Tortosa, Tarragona, España; Institut d´Investigació Sanitària Pere Virgili, Hospital Universitario Joan XXIII de Tarragona, España

**Keywords:** COVID-19, gestión de laboratorio, relación lactato deshidrogenasa (LDH)-y-leucocitos, prueba de diagnóstico molecular, herramientas predictivas, recursos técnicos

## Abstract

**Objetivos:**

La enfermedad por coronavirus 2019 (COVID-19) se ha extendido por todo el mundo, representando una grave amenaza para la salud mundial. En la lucha contra esta pandemia, los hospitales provinciales necesitan diagnosticar rápidamente a los pacientes con COVID-19 para evitar colapsar los servicios de urgencias. Sin embargo, la elevada demanda de pacientes con síntomas respiratorios agudos impide el envío rápido de los resultados de la prueba de referencia la rRT-PCR, para la identificación de neumonía por COVID-19-positiva. El objetivo principal de este artículo es la identificación de indicadores clínicos útiles para complementar las pruebas rRT-PCR y ayudar a controlar este brote.

**Métodos:**

Se analizaron parámetros hemáticos, de coagulación e inflamatorios en 309 pacientes con resultados de rRT-PCR negativos (128) y positivos (181). Se clasificó como positivos a aquellos pacientes con una prueba diagnóstica molecular positiva.

**Resultados:**

Se encontraron diferencias estadísticamente significativas en el recuento de leucocitos (WBC), recuento de neutrófilos, recuento de linfocitos y lactato deshidrogenasa (LDH). El cociente LDH/WBC aumenta el rendimiento diagnóstico, habiendo mostrado la mejor AUC (0,783) y sensibilidad (82%) así como el mejor porcentaje (80,5%) de pacientes COVID-19 correctamente identificados.

**Conclusiones:**

La combinación del cociente LDH/WBC junto con las características clínicas de la enfermedad podría resultar útil en el manejo de los pacientes y mejorar los recursos técnicos de los hospitales, especialmente en un escenario crítico en el que escasean los equipos y reactivos necesarios para realizar las rRT-PCR.

## Introducción

La enfermedad por coronavirus 2019 (COVID-19) se identificó por primera vez en el Centro de Control y Prevención de Enfermedades (CDC) de China a partir de una muestra de frotis faríngeo de pacientes con neumonía de origen desconocido [1], habiéndole asignado posteriormente la OMS el nombre de 2019-nCoV [2]. Desde diciembre de 2019, la COVID-19 se ha extendido por todo el mundo, representando una amenaza global debido a su elevada incidencia y nivel de infectividad. Hasta el 21 de mayo de 2020, un total de 5.121.639 personas se habían infectado, de las cuales 333.323 fallecieron [3]. En la lucha contra esta pandemia mundial, urge identificar marcadores clínicos y analíticos de predicción diagnóstica y evolución hacia formas graves y fatales de la enfermedad. Contar con estos predictores resulta esencial en algunos departamentos hospitalarios, como es en los servicios de urgencias, para la estratificación del riesgo, la optimización de la asignación de los pacientes en áreas específicas y la optimización de recursos humanos y técnicos durante la pandemia. De este modo, los parámetros analíticos se han mostrado útiles a la hora de discriminar entre los casos graves y no graves [4], [5], [6], así como para determinar el riesgo de mortalidad [7]. Los hospitales deben centrarse en identificar correctamente la enfermedad de COVID-19 e implementar medidas para impedir o retrasar el avance de la enfermedad. No obstante, la rápida intervención depende de los resultados de los laboratorios clínicos, siendo el método de referencia para el diagnóstico etiológico de la COVID-19 la prueba de rRT-PCR, con prolongados tiempos de respuesta (de media, se tarda entre 3 y 5 h en obtener los resultados). Además, los hospitales se enfrentan a diversas limitaciones, tales como conseguir la certificación de los laboratorios, el coste de los equipos, disponer de personal cualificado [8] y la capacidad para proporcionar al personal de laboratorio la protección adecuada, a la vez que se obtienen resultados fiables [9]. En este momento, el elevado volumen de pacientes con síntomas respiratorios agudos que llegan a los servicios de urgencias está evidenciando las limitaciones de las rRT-PCR. Este hecho, hace necesario el empleo de otras pruebas alternativas que permitan una rápida identificación de los pacientes infectados en el momento de su ingreso hospitalario. De esta manera, se favorecería el inicio precoz del tratamiento.

En Tarragona, provincia de Cataluña (España), ya se han realizado en los laboratorios clínicos 17.277 rRT-PCR a pacientes con sospecha de infección por coronavirus. El número de análisis se ha incrementado en más del 1.200% con respecto a un año normal sin COVID-19. El laboratorio se ha enfrentado a serias dificultades debido a la escasez de reactivos, lo que derivó en el colapso de algunos departamentos médicos, debido al retraso en la entrega de resultados. Los hospitales provinciales no disponen de suficientes áreas para ubicar a los pacientes sin diagnosticar, por lo que es importante poder contar con los resultados lo antes posible, con el fin de poder organizar los ingresos hospitalarios. En este escenario, urge hallar métodos diagnósticos accesibles.

El propósito de este estudio es desarrollar y validar la aplicación de herramientas predictivas que discriminen entre pacientes COVID-19 positivos y negativos, para prevenir la transmisión del virus en áreas hospitalarias y garantizar que se adoptan las medidas oportunas para el seguimiento y tratamiento de los pacientes. El uso combinado de potentes parámetros analíticos podría facilitar la gestión médica de los pacientes, optimizando los recursos humanos y materiales de los centros hospitalarios, que se han enfrentado a una importante escasez de equipos médicos y reactivos para poder realizar las rRT-PCR.

## Materiales y métodos

### Pacientes y obtención de muestras

En este estudio se incluyeron un total de 309 casos anonimizados mayores de 18 años (129 mujeres y 180 hombres), clasificados en dos grupos, basándonos en los resultados de la prueba de reacción en cadena de la polimerasa (rRT-PCR): con rRT-PCR negativa (128) y positiva (181). Se consideró que un caso era positivo si el gen *E* (gen de detección) era <35 ciclos (Ct) o si el gen *E* era >35 Ct con el gen *N* (gen confirmatorio) < 40 Ct. Si esto no era posible, se utilizó el *RdRp* como gen confirmatorio. La muestra la componían los pacientes admitidos en el Servicio de Urgencias del Hospital Universitario Joan XXIII (Tarragona, España) entre el 13 de marzo y el 21 de mayo de 2020 como casos potenciales de COVID-19 que presentaron fiebre y síntomas respiratorios iniciales [10].

En el análisis estadístico de este estudio únicamente se emplearon los datos anonimizados obtenidos a partir de análisis diagnósticos rutinarios. No se realizaron otros experimentos adicionales, por lo que no fue necesario obtener consentimiento informado. De este modo, este estudio está exento de aprobación por parte del Comité Ético local.

Se tomaron muestras de las vías altas introduciendo las muestras de frotis nasofaríngeo/orofaríngeo en tubos con medio de transporte universal de virus (DeltaSwab ViCUM^®^, Deltalab, Barcelona, España); así mismo, a los pacientes con enfermedad respiratoria más grave, se les tomaron muestras de las vías bajas: esputo (en su caso) y/o aspirado endotraqueal o lavado broncoalveolar, que se introdujo en tubos estériles (EUROTUBO^®^, Deltalab, Barcelona, España). Todas las muestras se tomaron siguiendo las recomendaciones de la OMS [11]. Las muestras de sangre se introdujeron en tubos con EDTA y sin anticoagulantes (Vacutainer, Becton Dickinson, Rutherford, NJ, EE. UU).

La sangre se analizaba el mismo día en que se realizó la rRT-PCR, incluyendo un hemograma completo en el analizador Sysmex XN-9000^®^ (Sysmex Corporation, Kobe, Japón), la determinación de la proteína C-reactiva (CRP), lactato deshidrogenasa (LDH), creatinina y ferritina, analizados con un sistema ADVIA^®^ Chemistry XPT (Siemens Healthcare Diagnostics Inc., Tarrytown, NY, EE. UU). Así mismo, la determinación de troponina I de alta sensibilidad (TNIH) y del péptido natriurético de tipo N-terminal pro-B (NT-proBNP) se realizó con un analizador ADVIA Centaur^®^ XPT Immunoassay (Siemens Healthcare Diagnostics Inc., Tarrytown, NY, EE. UU). El dímero D y el fibrinógeno se analizaron con el sistema ACL TOP 500 CTS^®^ (Werfen, Barcelona, España). La rRT-PCR se realizó con un termociclador CFX96 Touch System thermocycler (Bio-Rad Laboratories Inc., Hercules, California, EE.UU) empleando un kit comercial dirigido a los genes *E*, *N* y *RdRp* (LightMix^®^ Modular SARS and Wuhan CoV, TIB MOLBIOL, Berlin, Alemania). La purificación de ARN se realizó con el mini kit RN easy en el analizador QIAcube Connect (QIAGEN, Hilden, Alemania).

### Análisis estadístico

Los datos cuantitativos con distribución normal se expresan como medias ± DE. Los datos sin distribución normal se expresan como medianas y rangos intercuartílicos (IQR). El estudio comparativo entre grupos se realizó con la prueba t de Student. Los datos sin distribución normal se analizaron con el test U de Mann-Whitney. Las variables categóricas se compararon con el test exacto de Fisher. Para analizar las diferencias entre los resultados de las rRT-PCR según los factores demográficos de los pacientes, se empleó el análisis de varianza unidireccional (ANOVA) para las variables con más de dos categorías (rango de edad).

La precisión diagnóstica de cada parámetro analítico se evaluó mediante el índice de Youden (YI), sensibilidad, especifidad, y valores predictivos positivos y negativos, que se calcularon de la siguiente manera: YI=sensibilidad+especifidad–100; sensibilidad=verdadero positivo/(verdadero positivo+falso negativo); especifidad=verdadero negativo /(verdadero negativo+falso positivo); valor predictivo positivo=verdadero positivo/(verdadero positivo+falso positivo); valor predictivo negativo=verdadero negativo/(verdadero negativo+falso negativo). El índice de Youden es una función de la sensibilidad y especifidad, que depende de las distribuciones de las poblaciones infectadas (esto es, rRT-PCR-positivas) y no infectadas (rRT-PCR-negativas), así como de las medidas de efectividad de un marcador diagnóstico, lo que permite identificar un valor de corte óptimo para el marcador [12]. Además, se construyeron curvas características operativas del receptor (ROC) para calcular el área bajo la curva (AUC) de cada índice.

Los datos obtenidos se procesaron con el programa SPSS versión 25.0 para Windows (SPSS, Chicago, IL, EE. UU). Un valor p de <0,05 se consideró estadísticamente significativo.

## Resultados

Este estudio incluye los parámetros analíticos de 309 pacientes anonimizados, con 128 rRT-PCR negativas (mediana de edad: 69 años, rango: 20–69) y 181 resultados de rRT-PCR positivos (mediana de edad 70 años, rango: 19–93). Los datos demográficos de los dos grupos se muestran en la Tabla 1. No se observaron diferencias significativas en relación con la edad entre el grupo de positivos y negativos (p=0,751). Sin embargo, si comparamos los rangos de edad, se observaron diferencias significativas con respecto al Ct de los genes *E* (p <0,0001) y *N* (p=0,002) detectados en el grupo de rRT-PCR positivos. Los pacientes mayores de 70 años mostraron menos Ct que los pacientes en otros rangos de edad. Además, hubo diferencias significativas (p=0,027) con respecto al sexo entre el grupo de positivos (115 hombres, 63.5%) y negativos (65 hombres, 50,8%).

**Tabla 1:** Características demográficas de los pacientes admitidos en el Servicio de Urgencias del Hospital Universitario Joan XXIII (del 13 de marzo al 21 de mayo de 2020).

	Grupo con rRT-PCR positiva^a^ (n=181)				Grupo con rRT-PCR negativa^b^ (n=128)	Valor p de significación (entre^a-b^)
		Gene, Ct^d^				
		*E* (n=181) Media ± DE	*R* (n=81) Media ± DE	*N* (n=114) Media ± DE		
**Edad**, años	70 (19–93)				69 (20–96)	0,751
**Rango** ^c^	19–93				20–96	
≤39	13 (7,2%)	30,20 ± 7,68	29,02 ± 9,33	31,63 ± 6,60	13 (10,2%)	
40–49	17 (9,4%)	31,03 ± 5,05	33,83 ± 1,75	34,32 ± 3,06	17 (13,3%)	
50–59	35 (19,3%)	30,60 ± 4,93	30,02 ± 5,18	31,70 ± 6,78	16 (12,5%)	
60–69	22 (12,2%)	26,97 ± 6,52	29,40 ± 5,21	30,36 ± 5,41	21 (16,4%)	
≥70	94 (51,9%)	25,02 ± 6,28	27,31 ± 6,15	27,53 ± 6,35	61 (47,7%)	
**Valor p (entre** ^**c-d**^ **)**		<0,0001^e^	0,061	0,002^e^		
**Sexo**						0,027^e^
Mujer	66 (36,5%)				63 (49,3%)	
Hombre	115 (63,5%)				65 (50,8%)	

DE, desviación estándar; Ct, umbral de ciclo. Como la edad no presentaba una distribución normal, esta se expresa como mediana y rango. Se utilizó el test de Mann–Whitney para comprobar si había diferencias estadísticamente significativas entre el grupo con rRT-PCR positiva^a^ y negativa^b^. Se aplicó el análisis de varianza unidireccional (ANOVA) para comparar el rango de edad^c^ y la detección de genes mediante rRT-PCR^d^. En cuanto al sexo, se aplicó el test de Fisher para comprobar si existía alguna asociación entre los pacientes con rRT-PCR positiva^a^ y negativa^b^. ^e^indica valores p inferiores a 0,05.

En el momento del ingreso en Urgencias, se realizó un hemograma completo y una rRT-PCR a todos los pacientes con síntomas iniciales de infección de COVID-19. En la Tabla 2 se muestran todos los parámetros analíticos estudiados. El grupo con rRT-PCR positiva mostró un descenso significativo en el recuento de leucocitos (p <0,0001), neutrófilos (p <0,0001), linfocitos (p <0,0001) y plaquetas (p =0,045), frente al grupo de pacientes negativos. Además, los pacientes con rRT-PCR positiva mostraron elevados niveles de lactato deshidrogenasa (p <0,0001), frente a los pacientes negativos. Sin embargo, no se detectaron diferencias en el recuento de glóbulos rojos, la coagulación (dímero D y fibrinógeno), TNIH y NT-proBNP.

**Tabla 2:** Comparación de los parámetros analíticos entre el grupo con rRT-PCR positiva y negativa.

Parámetros analizados	Grupo con rRT-PCR positiva^a^ (181)		Grupo con rRT-PCR negativa^b^ (n=128)		Valor p de significación (entre^a-b^)
	Media ± DE		Media ±DE		
**Hemograma rutinario**					
Glóbulos rojos (RBC), ×10^6^/µL	n=176	4,16 ± 0,84	n=128	4,11 ± 0,76	0,846
Hemoglobina(Hb), g/dL	n=176	12,11 ± 2,13	n=128	12,03 ± 2,22	0,544
Hematocrito (Htc), %	n=176	37,29 ± 6,45	n=128	37,30 ± 6,39	0,809
Volumen celular medio (MCV), fL	n=176	90,44 ± 7,43	n=128	91,22 ± 7,78	0,780
Hemoglobina corpuscular media (MCH), pg	n=176	29,31 ± 2,65	n=128	29,38 ± 2,88	0,648
Concentración media de hemoglobina corpuscular (MCHC), g/dL	n=176	32,38 ± 1,42	n=128	32,19 ± 1,40	0,481
Ancho de distribución de glóbulos rojos (RDW), %	n=176	14,12 ± 2,28	n=128	14,34 ± 1,97	0,474
	**Mediana (IQR)**		**Mediana (IQR)**		
Leucocitos (WBC), ×10^9^/µL	n=176	6,50 (24,99)	n=128	9,27 (23,50)	<0,0001^c^
Neutrófilos, ×10^9^/µL	n=176	4,89 (21,44)	n=128	6,76 (23,32)	<0,0001^c^
Linfocitos, ×10^9^/µL	n=176	1,01 (4,44)	n=128	1,38 (3,05)	<0,0001^c^
Plaquetas, ×10^9^/µL	n=176	201,5 (547)	n=128	223,5 (739)	0,045^c^
**Coagulación**					
Dímeros D, ng/dL	n=121	715,0 (67976)	n=26	520,50 (14012)	0,391
Fibrinógeno, mg/dL	n=154	672,0 (1117)	n=107	631,0 (649)	0,074
**Bioquímica**					
Proteína C reactiva, mg/dL	n=164	6,45 (35)	n=125	6,30 (50,60)	0,519
Creatinina, mg/dL	n=174	0,83 (13,19)	n=126	0,88 (5,01)	0,810
Lactato deshidrogenasa, U/L	n=139	248 (763)	n=58	209,50 (370)	<0,0001^c^
Ferritina, ng/mL	n=23	672,0 (4567)	n=7	249,0 (403)	0,077
Troponina I de alta sensibilidad (TNIH), ng/L	n=103	10,0 (5218)	n=33	13,0 (23803)	0,092
Péptido natriurético de tipo N-terminal pro-BNP (NT-proBNP), pg/mL	n=14	642 (9224)	n=21	2683,0 (27312)	0,069

DE, desviación estándar; IQR, rango intercuartílico. Se realizó la prueba t de Student en los siguientes parámetros: glóbulos rojos, hemoglobina, hematocrito, VCM, HCM, CHCM y ancho de distribución de glóbulos rojos. Se realizó el test U de Mann-Whitney en los siguientes parámetros: leucocitos, neutrófilos, linfocitos, plaquetas, dímeros D, fibrinógeno y bioquímica. Se emplearon las dos pruebas estadísticas para comprobar si había diferencias estadísticamente significativas entre el grupo de pacientes con rRT-PCR positiva^a^ y negativa^b^. ^c^indica valores p inferiores a 0,05.

**Figura 1:**
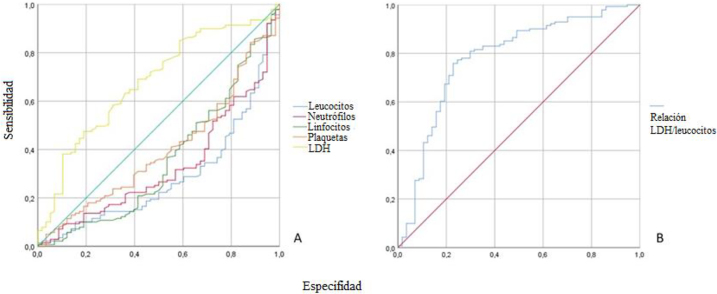
Curvas ROC de los parámetros analíticos (A) y cociente LDH/WBC (B) para diferenciar al grupo con rRT-PCR positiva frente a rRT-PCR negativa.

De este modo, en virtud de las diferencias significativas observadas entre el grupo de pacientes positivos y negativos, se calcularon la AUC, los valores umbral, la sensibilidad, especifidad, y el índice de Youden de los parámetros analíticos eficientes (Tabla 3). En la Figura 1A, la mayor AUC se obtuvo con los leucocitos y la LDH (0,713 y 0,679, respectivamente). Además, el recuento de leucocitos fue el parámetro con mayor sensibilidad (76%) de entre todos los examinados. La LDH fue la que mostró mayor especifidad (81%). Basándonos en estos resultados, probamos una nueva relación LDH/leucocitos (Tabla 3). Los valores de corte superiores a 25,64 indicaron positividad. La relación LDH/leucocitos fue la herramienta más efectiva, teniendo la mayor AUC (0,783, Figura 1B) y sensibilidad (82%), frente a otros parámetros significativos. La nueva relación LDH/leucocitos también fue la que obtuvo un mayor porcentaje de pacientes correctamente identificados (80,5%).

**Tabla 3:** Precisión diagnóstica de los parámetros significativos empleados en este estudio y valores de corte para discriminar a los pacientes con rRT-PCR positiva de aquellos con resultado negativo.

Parámetros	AUC (IC95%)	Valor de corte del grupo de pacientes positivos	S, %	E, %	VPP, %	VPN, %	PC, %	Índice de Youden’s, %
Leucocitos	0,713 (0,635–0,791)	<7,24	76	63	71	81	76	39
Neutrófilos	0,674 (0,591–0,758)	<5,25	69	65	71	77	74	34
Linfocitos	0,643 (0,556–0,730)	<1,48	50	77	81	65	73	27
Plaquetas	0,602 (0,518–0,686)	<208,0	69	53	68	71	69,5	22
Lactato deshidrogenasa (LDH)	0,679 (0,598–0,760)	>260,50	48	81	66	83	74,5	29
LDH/leucocitos	0,783 (0,707–0,856)	>25,64	82	69	85	76	80,5	51

AUC, área bajo la curva; IC, intervalos de confianza; S, sensibilidad; E, especifidad; VPP, valor predictivo positivo (%); VPN, valor predictivo negativo (%); PC, pacientes identificados correctamente (%).

## Discusión

El coronavirus humano es uno de los principales patógenos de infección respiratoria. La COVID-19 suele progresar rápidamente a neumonitis e incluso a síndrome de dificultad respiratoria aguda y causar la muerte [13]. En este estudio se incluyó a todos los pacientes que se presentaron en el servicio de urgencias (309 pacientes) con síntomas compatibles con la COVID-19. Se realizaron las rRT-PCR a todos los pacientes para clasificarlos como positivos o negativos. Hubo una mayoría de hombres (63.5%) en el grupo de positivos (n=181). Li et al. [14] y Guan et al. [15] también observaron un mayor índice de infección entre la población masculina. Nanshan et al. [16] atribuyeron la menor susceptibilidad de las mujeres a la infección vírica a la protección del cromosoma X y de las hormonas sexuales, que juegan un importante papel en la inmunidad innata y adaptativa. A decir verdad, la COVID-19 ha evidenciado la vulnerabilidad de la población envejecida a las enfermedades emergentes [17]. El 51,9% de los pacientes con rRT-PCR positiva eran mayores de 70 años y fueron los que presentaron menores valores de Ct para los genes *E*, *R* y *N*, frente a otros rangos de edad. Al parecer, la carga viral (inversamente proporcional al valor Ct) podría estar relacionada con la gravedad y el pronóstico de la infección de COVID-19 [18].

El propósito de este estudio era desarrollar y validar la aplicación de herramientas predictivas capaces de discriminar entre pacientes con y sin COVID-19, con el fin de obtener los resultados lo antes posible para poder organizar las admisiones y prevenir la transmisión del virus en las áreas hospitalarias cuando se retrasan los resultados de la rRT-PCR. En cuanto a las pruebas analíticas, el recuento de leucocitos, neutrófilos y linfocitos es significativamente inferior en los pacientes con rRT-PCR positiva. En estudios anteriores, los pacientes positivos presentaron un recuento bajo de leucocitos y linfocitos [19], [20], [21], lo que concuerda con nuestro estudio, no ocurriendo lo mismo con el recuento de neutrófilos [16], [19]. Además, el recuento de linfocitos podría servir como predictor clínico de infección, debido a su papel en la eliminación de células infectadas por virus. Las partículas virales de la COVID-19 dañan el componente citoplasmático de los linfocitos, destruyéndolos [22] y comprometiendo la capacidad del organismo para reponer dichas células [23]. Del mismo modo, tras una infección vírica, se entiende que la linfopenia en sangre periférica se produce por el secuestro de linfocitos [24]. Xiong et al. [25] explica que el quimioatrayente de neutrófilos CXCL2 y CXCL8 facilita la migración de estas células inmunes al lugar de la infección. Al parecer, la neutropenia observada en los pacientes positivos se debe a la infiltración celular en el tejido pulmonar de los pacientes con COVID-19. Con respecto a la coagulación, se observaron diferencias estadísticamente significativas en el recuento de plaquetas entre los dos grupos. La trombocitopenia observada en el momento de ingreso hospitalario en los pacientes con COVID-19 también fue identificada por Liu et al. [13] como un predictor pronóstico. El consumo de plaquetas está asociado a una elevación del dímero D y del fibrinógeno [13], [26]. En este estudio, el grupo con rRT-PCR positiva mostró mayores niveles de dímero D y fibrinógeno, frente al grupo con rRT-PCR negativa, aunque la diferencia no fue significativa. Basándonos en los hallazgos inflamatorios y de daño orgánico, la LDH estaba significativamente elevada en el grupo con rRT-PCR positiva. Este valor ha demostrado ser una herramienta eficaz a la hora de discriminar entre los pacientes infectados y sin infección de COVID-19 [8] y predecir la gravedad de la enfermedad [27]. No se observaron diferencias significativas en PCR o ferritinina, lo que contrasta con los resultados de estudios anteriores [8], [19].

Se podría desarrollar un método predictivo basado en análisis de sangre rutinarios, ya que el recuento de leucocitos, neutrófilos y linfocitos y la LDH mostraron una buena precisión a la hora de predecir los casos con rRT-PCR positiva. El recuento de leucocitos y la LDH fueron los parámetros que mostraron mayor AUC (0,713 y 0,679), respectivamente), lo que sustenta el uso de la relación LDH/leucocitos, con la consiguiente mejora de la AUC (0,783) y de la sensibilidad (82%). Esta relación mostró un elevado porcentaje de identificación de positivos (80,5%) antes de realizar la rRT-PCR. Teniendo en cuenta la relación LDH/leucocitos, junto con signos clínicos compatibles con la COVID-19 en el momento del ingreso, se podría emplear un valor de corte superior a 25,64 como herramienta predictiva para clasificar a los pacientes con y sin infección y facilitar la gestión de los pacientes en los hospitales provinciales. Además, el resultado de la relación LDH/leucocitos puede ayudar a priorizar a los pacientes que necesitan hacerse una rRT-PCR de urgencia, de aquellos que pueden esperar, mejorando así el empleo de los recursos técnicos del laboratorio clínico. Sin embargo, la información de la que se dispone actualmente sobre valores de predicción temprana para los casos positivos es relativamente limitada y se requieren más estudios.

Este estudio presenta algunas limitaciones. En primer lugar, este es un estudio retrospectivo unicéntrico, por lo que se necesita una validación externa con estudios multicéntricos en los que se emplee un mayor número de muestras. En segundo lugar, se extrajeron datos analíticos sin consultar el historial médico de los pacientes y sin tener en cuenta sus comorbilidades o cualquier otra enfermedad crónica concomitante. La relación LDH/leucocitos se ajustaría mejor si se conocieran las patologías de los pacientes. En futuros estudios, sería interesante establecer diferentes puntos de corte, dependiendo de las características clínicas de los pacientes. Además, algunos casos con rRT-PCR negativa se pueden identificar erróneamente, debido a que la carga viral depende del tipo de muestra recogida y de la gravedad de los síntomas [28], [29]. Finalmente, el tamaño de la muestra fue relativamente pequeño, lo que puede influir en los resultados estadísticos.

## Conclusiones

Muchos estudios demuestran que algunos parámetros analíticos pueden discriminar los casos graves de los leves o determinar el riesgo de mortalidad en los pacientes con COVID-19. Sin embargo, la rRT-PCR sigue siendo el método de referencia más fiable para distinguir los casos de neumonía COVID-19 positivos de los negativos, aunque el proceso es lento y requiere el uso de equipos ya de por sí limitados. Este estudio se centra en la gestión de los recursos técnicos en época de pandemia, cuando el número de pacientes infectados aumenta notablemente y el rendimiento de las rRT-PCR se ve limitado por la escasez de reactivos y de personal de laboratorio. En este escenario crítico, la relación LDH/leucocitos combinada con síntomas compatibles con la COVID-19 sería una buena herramienta para predecir qué pacientes tienen más probabilidad (80,5%) de presentar la enfermedad. De este modo, la relación LDH/leucocitos podría ayudar a priorizar las rRT-PCR para aquellos pacientes infectados y mejorar la asistencia en los servicios de urgencias colapsados por una elevada carga asistencial.
